# Renal disorder and biological treatment in juvenile idiopathic arthritis

**DOI:** 10.1186/1546-0096-12-S1-P342

**Published:** 2014-09-17

**Authors:** Lidia Mateescu, Mariana Stefan

**Affiliations:** 1 "Regina Maria" The Private Healthcare Network, Bucharest, Romania

## Introduction

The paper will present and discuss the renal complications occurred after two years of biological treatment in the case of an 8- year old female patient, who was previously diagnosed with a poliarticular form of Juvenile Idiopathic Arthritis.

## Objectives

The authors take into discussion the occurred kidney disorder as a side effect of the biological treatment.

## Methods

Long term follow up of the patient contained periodical clinical examination and laboratory investigation. Imagistic methods and renal biopsy were also needed in the evaluation process.

## Results

The patient was diagnosed with Juvenile Idiopathic Arthritis when she was 3 years and 2 months old, for which she was first treated with nonsteroidal antiinflamatory drugs, then DMARDS (Methotrexate), but without a very good course of the disease. When she reached the age of 4 years and 5 months she started biological treatment, with a good response clinically and of the laboratory tests. After two years from the initiation of this therapy, the patient developed signs of kidney injury. To elucidate the etiology of renal impairment, biological and imagistic investigations were conducted in dynamic. After that, renal biopsy established the diagnosis. The biological treatment was stopped and she started therapy for kidney disorder, with a favorable response in a few months. Currently the patient is still under medical observation for the initial disease.

## Conclusion

The occurrence of renal impairment is discussed as a side effect of the biological treatment. Similar, still rare references can be found in medical literature.

## Disclosure of interest

None declared

